# Supplementary material for: Epididymal epithelium propels early sexual transmission of Zika virus in the absence of interferon signaling
Source: Nat Commun. 2021 Apr 29;12:2469. doi: 10.1038/s41467-021-22729-5 (PMC8084954; doi:10.1038/s41467-021-22729-5)
Supplement: Supplementary file 1 — Supplementary Information [file 41467_2021_22729_MOESM1_ESM.pdf]

Supplementary information: Figures and Tables

Epididymal epithelium propels early sexual transmission of Zika virus in the absence of interferon signaling

Pletnev et al.

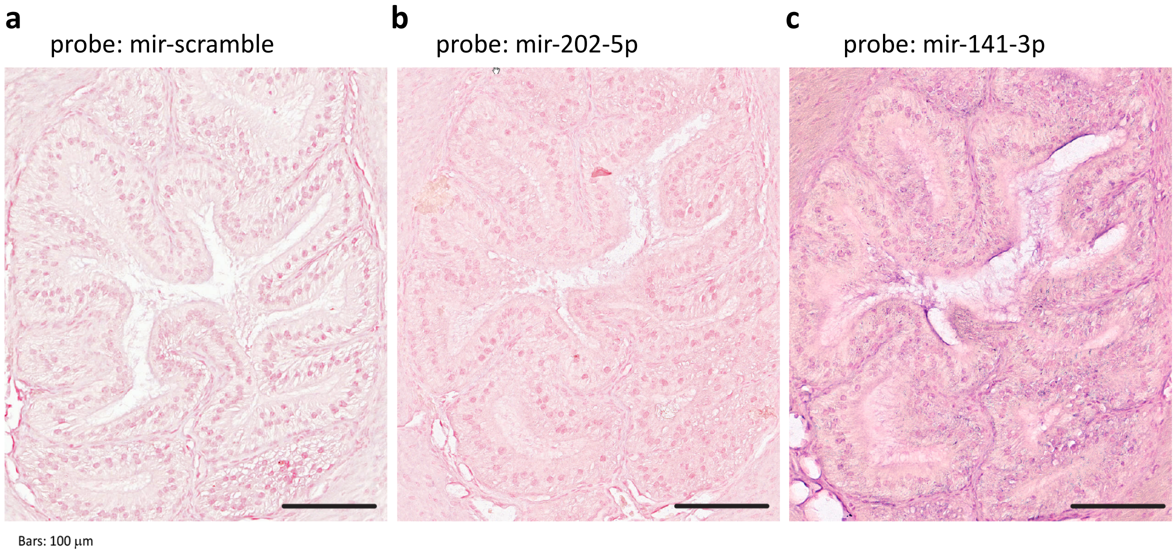

Figure S1. Detection of mir-202-5p and mir-141-3p in the vas deferens of AG129 mice by *in situ* hybridization.
In situ hybridization of the epithelial cells of vas deferens using (**a**) mir-scramble probe (negative control), (**b**) RNA probe complementary to miRNA mir-202-5p and (**c**) RNA probe complementary to mir-141-3p. Each micrograph shows a presentative image of indicated miRNA expression in the vas deferens (number of mice = 3; excrement was performed once). Scale bars: 100 µm.

a

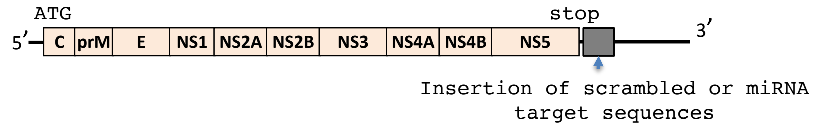

b

ZIKV-NS3m

stop ⇓ 10 ⇓ 20

taagcaccaatcttaatgttgtc

2×scr

scr NsiI scr

taagcaccaatCAGCACGACTTCTTCAAGTC*atgcat*accaatcttaatCAGCACGACTTCTTCAAGTCtgttgtc

2×202(T)

mir-202-5p(T) XhoI mir-202-5p(T)

taagcaccaatCAAAGAAGTATATGCATAGGAA*ctcgag*accaatcttaatCAAAGAAGTATATGCATAGGAAtgttgtc

2×141(T)

mir-141-3p(T) NsiI mir-141-3p(T)

taagcaccaatCCATCTTTACCAGACAGTGTTA*atgcat*accaatcttaatCCATCTTTACCAGACAGTGTTAtgttgtc

2×202/141(T)

mir-202-5p(T) mir-141-3p(T) XhoI mir-202-5p(T) ClaI

taagcaccaaCAAAGAAGTATATGCATAGGAACCATCTTTACCAGACAGTGTTA*ctcgag*CAAAGAAGTATATGCATAGGAA*ATCGAT*t

mir-141-3p(T)

CCATCTTTACCAGACAGTGTTAatgttgtc

ZIKV-lib/n11

n11 region NsiI scr

taagcaccaatCAGCACGACNNNNNNNNNNN*atgcat*accaatcttaatCAGCACGACTTCTTCAAGTCtgttgtc

Figure S2. Viruses used in the study.

(**a**) Schematic representation of ZIKV genome following insertion of scr sequences, miRNA targets, or sequences serving as unique molecular identifiers (gray box). (**b**) The 5' terminus of the 3'NCR of ZIKV-NS3m, 2×scr, 2×202(T), 2×141(T), 2×202/141(T), and ZIKV-lib/n11 viruses containing scr or miRNA targets insertion. Arrows and underlined nucleotides highlight positions of sequence insertions. Sequence highlighted in yellow indicates stop codon of ZIKV open reading frame. NsiI, XhoI and ClaI - engineered sites for restriction endonucleases that were used for construction of the clones.

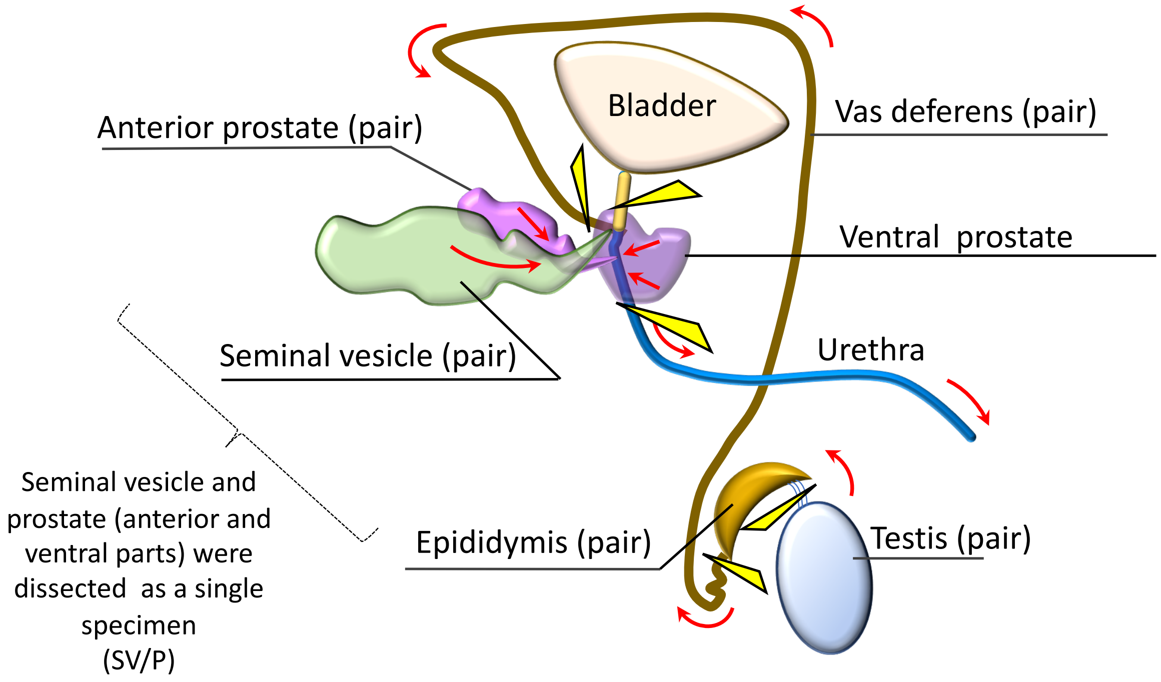

Figure S3. Dissection of the organs within the MRS.
Schematic representation of the MRS, showing natural flow of sperm or fluids produced by accessory glands (red arrows). Yellow triangles show locations of cuts made to separate specific parts of the MRS, which were analyzed in this study.

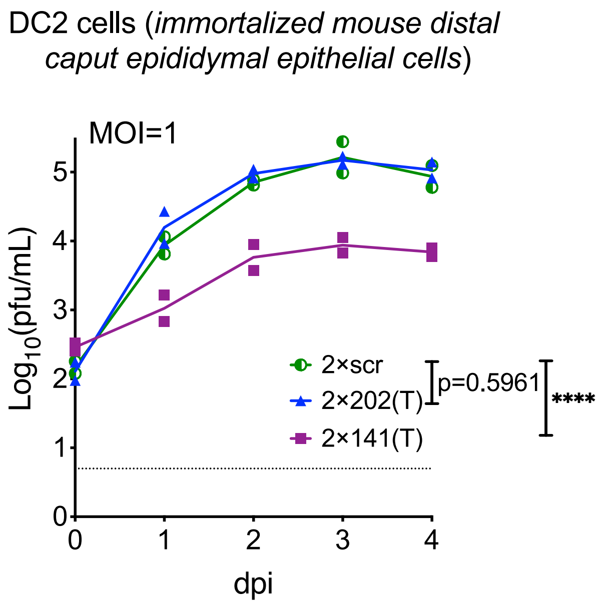

Figure S4. Growth of 2×scr, 2×202(T), and 2×141(T) viruses in DC2 cells.
Six-well plates were coated with Applied Cell Extracellular Matrix (1 mL/well; cat# G422, ABM). Two million DC2 cells (immortalized mouse distal caput epididymal epithelial cells; cat# T059, Applied Biological Materials Inc) were seeded into each well of the plate in 3 mL of complete PriGrow medium [PriGrow medium (Cat# MT015, ABM), 10% FBS, 1% Penicillin/Streptomycin solution (Cat# G255, ABM)]. The next day, cells were infected with 2×scr, 2×202(T), or 2×141(T) viruses in duplicate wells at an MOI of 1 for 1 hour at 33°C, 5% CO2. Cells were washed twice with 3 mL of complete PriGrow medium and maintained in 3mL of complete PriGrow medium at 33°C, 5% CO2. Aliquots of cells supernatant (0.3 mL) were collected daily and stored at -80°C, and each time culture supernatants were replenished with 0.3 mL of complete PriGrow medium. Samples were titrated by plaque assay in Vero cells. Differences in virus growth kinetics were compared using 2-way ANOVA (we compared the mean titer for all time points determined for each of the viruses) with multiple comparison adjustment (Dunnett's test) implemented in Prism 8 software (GraphPad Software, San Diego, CA). The dashed line indicates the limit of virus detection: 0.7 log_{10}(pfu/mL). **** indicates $p<0.001$

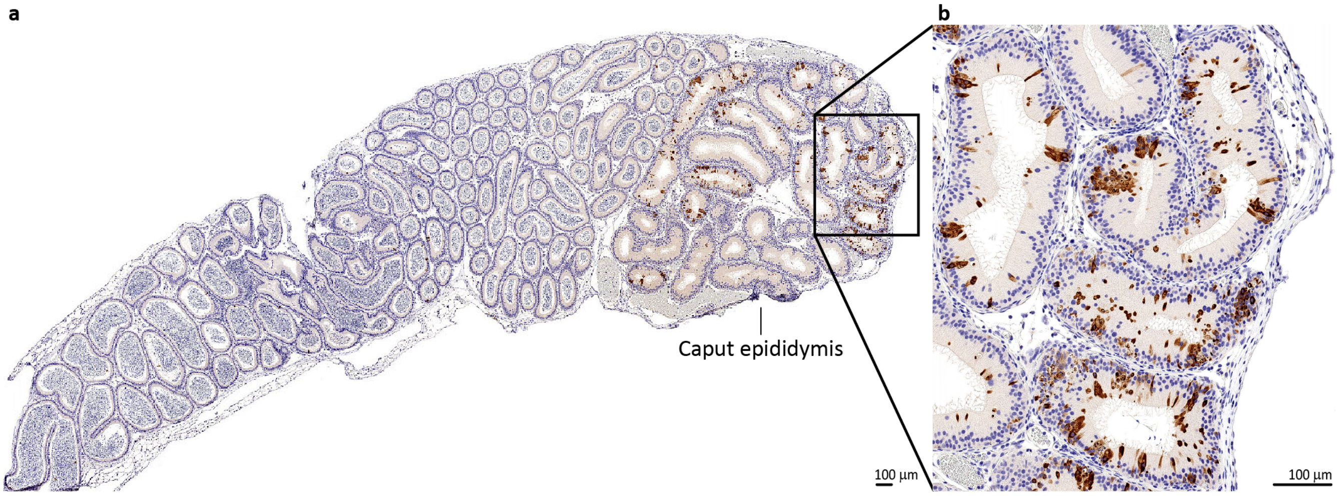

Figure S5. Infection of the epididymal epithelium by putative escape mutants of 2×141(T) virus. Adult AG129 mice (n=4) were infected IP with 10^6 pfu of 2×141(T) virus and sacrificed at 9 dpi as described in the legend for Fig. 2 (excrement was performed once). ZIKV antigen positive cells were detected in one out of 8 tested epididymides (see Fig. 2m), which is depicted in this figure. (**a**) overview of the epididymis showing positive ZIKV-IR (brown) in the caput epididymis. (**b**) boxed area in (**a**) is shown at higher magnification and illustrates multiple ZIKV+ cells of the epididymal epithelium (brown). Scale bars: 100 mm.

a

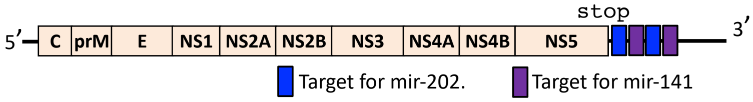

b

Mating pair #1

Escape mutant#1 (Esc1) was found in the female serum:

stop mir-202-5p(T) mir-141-3p(T) XhoI mir-202-5p(T) ClaI mir-141-3p(T)
TAAG~~CACCAAcaaagaagtatatgcataggaaccatctttaccagacagtgttaCTCGAGcaaagaagtatatgcataggaaATCGATtccatcttt~~accagacagt
gttaaTGTTGTCAGGCCTGCTAGTCAGCCACAGCTTGGGGAAAGCTGTGCAGCCTGTGACCCCCCCAGGAGAAGCTGGGAAACCAAGCCTATAGTCAGGCCGAGAAC

Escape mutant#2 (Esc2) was found in the epididymis:

TAAGCACCAAcaaagaagta~~tatgcataggaaccatctttaccagacagtgttaCTCGAGcaaagaagtatatgcataggaaATCGATtccatctttaccagacagt~~
~~gttaaTGTTGTCAGGCCTGCTAGTCAGCCACAGCTTGGGGAAAGCTGTGCAGCCTGTGACCCCCCCAGGAGAAGCTGGGA~~AACCAAGCCTATAGTCAGGCCGAGAAC

Mating pair #2

Escape mutant#3 (Esc3) was found in the female serum, epididymis and vas deferens:

TAAGCACCAAcaaagaagtatatgcat~~aggaaccatctttaccagacagtgttaCTCGAGcaaagaagtatatgcataggaaATCGATtccatctttaccagacagt~~
~~gttaaTGTTGTCAGGCCTGCTAGTCAGCCACAGCTTGGGGAAAGCTGTGCAGCCTGTGACCCCCCCAGGAGAAGCTGGG~~AAACCAAGCCTATAGTCAGGCCGAGAAC

Escape mutant#4 (Esc4) was found in the FRT and in the epididymis and vas deferens:

TAAGCACCAA~~caaagaagtatatgcataggaaccatctttaccagacagtgttaCTCGAGcaaagaagtatatgcataggaaATCGATtccatcttt~~accagacagt
gttaaTGTTGTCAGGCCTGCTAGTCAGCCACAGCTTGGGGAAAGCTGTGCAGCCTGTGACCCCCCCAGGAGAAGCTGGGAAACCAAGCCTATAGTCAGGCCGAGAAC

Mating pair #3

Escape mutant#5 (Esc5) was found in the female serum, epididymis, vas deferens and SV/P:

TAAGCACCAAcaaagaagtatatgcataggaaccatctttacca~~gacagtgttaCTCGAGcaaagaagtatatgcataggaaATCGATtccatcttta~~ccagacagt
gttaaTGTTGTCAGGCCTGCTAGTCAGCCACAGCTTGGGGAAAGCTGTGCAGCCTGTGACCCCCCCAGGAGAAGCTGGGAAACCAAGCCTATAGTCAGGCCGAGAAC

Figure S6. Escape mutants (Esc) in the genome of 2×202/141(T) virus isolates from female serum and from various organs of the MRS of male mating parents.
(**a**) Schematic representation of 2×202/141(T) virus genome. (**b**) Escape mutants carrying deletions in the 3′NCR of 2×202/141(T) virus detected in male and female AG129 mice. Experimental design and outcome of the 2×202/141(T) virus transmission study are summarized in Fig. 3. Each deletion (Esc) in the 3′NCR of 2×202/141(T) is represented by a strikethrough sequence.

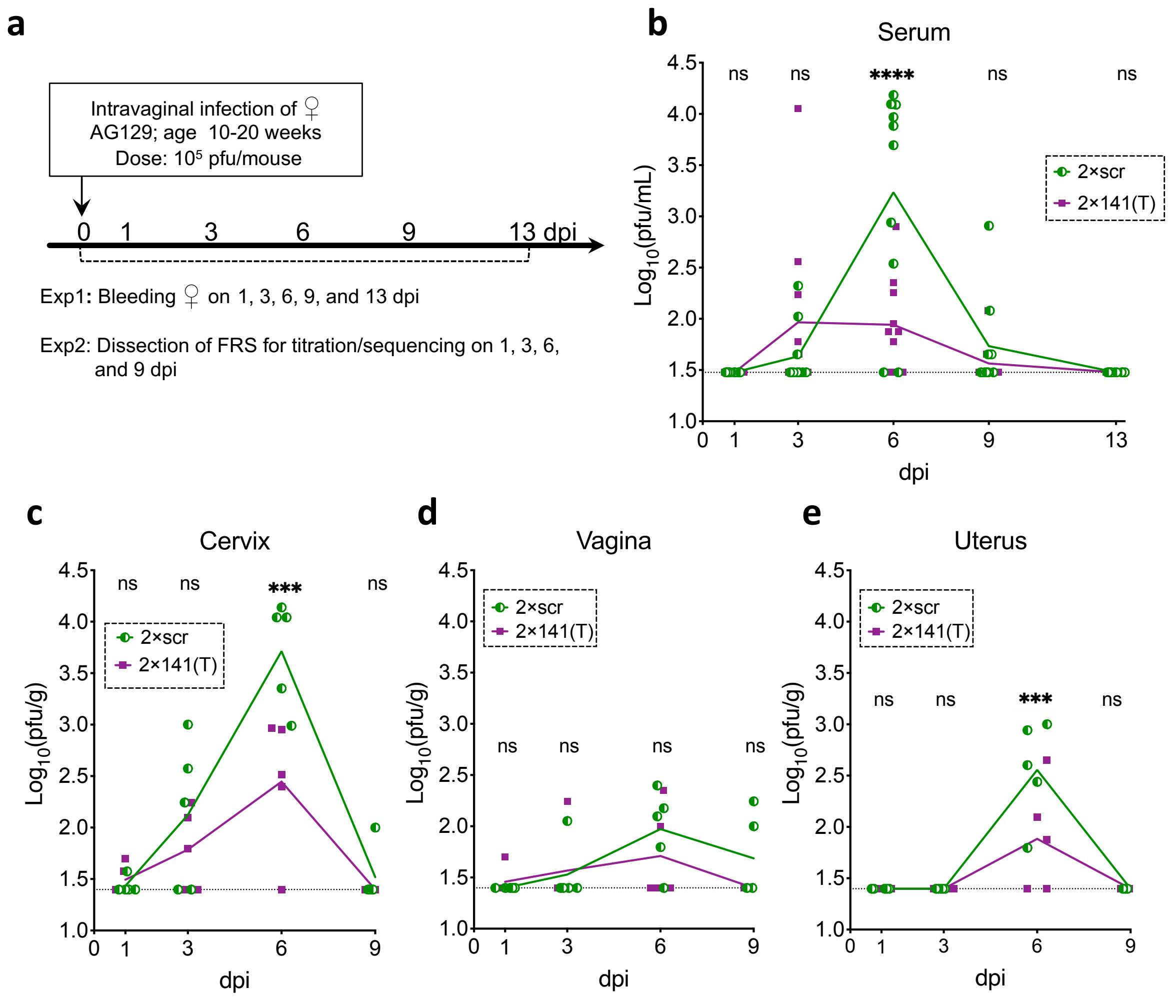

Figure S7. Replication of 2×scr and 2×141(T) in the serum and organs of the FRS of AG129 mice after intravaginal infection.

(**a**) Experimental design of the study. (**b**) Ten AG129 mice were infected intravaginally with 10^5 pfu of 2×scr and 2×141(T) viruses. At 1, 3, 6, 9, and 13 dpi mice were bled to determine viremia. Mice were kept in the cages until 28 dpi for evaluation of serocoversion rate by PRNT50 test (See Fig. 3c).(**c-e**) Twenty AG129 mice were infected intravaginally with 10^5 pfu of 2×scr and 2×141(T) viruses. At 1, 3, 6, and 9 dpi four mice from each group were sacrificed. Mean viral titer in the cervix (**c**), vagina (**d**) and uterus (**e**) was determined by titration in Vero cells. The dashed lines indicate the limit of virus detection: 1.5 $\log_{10}$(pfu/mL) for serum (**b**) and 1.4 (pfu/g) for mouse organs (**c-e**). Differences between the titer of 2×scr and 2×141(T) viruses in the serum and mouse organs were compared using two-way ANOVA with multiple comparison adjustment (Sidak's test). p values (from left to right): (**b**) ns, p=>0.9999; ns, p=0.5060; ns, p=0.9504; p=>0.9999. (**c**) ns, p=0.9990; ns, p=0.6255; ns, p=0.9859. (**d**) ns, p=0.9966; ns, p=0.9994; ns, p=0.5586; ns, p=0.4701. (**e**) ns, p>0.9999; ns, p>0.9999; ns, p=>0.9999. (*** p<0.001; **** p< 0.0001).

Experiment description:

We showed that after intravaginal inoculation the infectivity of 2×scr and 2×141(T) viruses to AG129 female mice was identical (Fig. 3c). It is possible that inefficient sexual transmission of the 2×141(T) virus might be attributed to its attenuated vaginal infection and replication in the FRS. To account for this possibility, we infected AG129 female mice with 2×scr and 2×141(T) viruses intravaginally and compared kinetics of virus replication in the serum and organs of FRS (Fig. S7a). At 6 dpi, the load of 2×141(T) virus in the cervix, uterus and in the serum was significantly reduced as compared to that of 2×scr virus (Fig. S7b, S7c, S7e**)**. This suggested that reduced transmission of 2×141(T) virus from infected male mice (Fig. 3b) could have been attributed (at least partially) to the attenuation of 2×141(T) virus replication in the female mice. However, growth kinetics of the viruses were indistinguishable in the vagina (Fig. S7d). Moreover, escape mutants were not detected in the 2×141(T) virus isolated from female serum or from organs of FRS. The precise anatomical routes of ZIKV progression from the ejaculate deposited into the FRS to the female serum remains poorly characterized. Therefore, the observed discrepancies in the effects of mir-141 targeting on ZIKV infectivity to different parts of FRS and on seroconversion rate of female mice (Fig. 3c) preclude us from making a definitive conclusion regarding the role of mir-141 targets on infectivity of 2×141(T) virus to female mice. This prompted us to perform the semen shedding studies, which allow us to omit the female infection step altogether (Fig. 4).

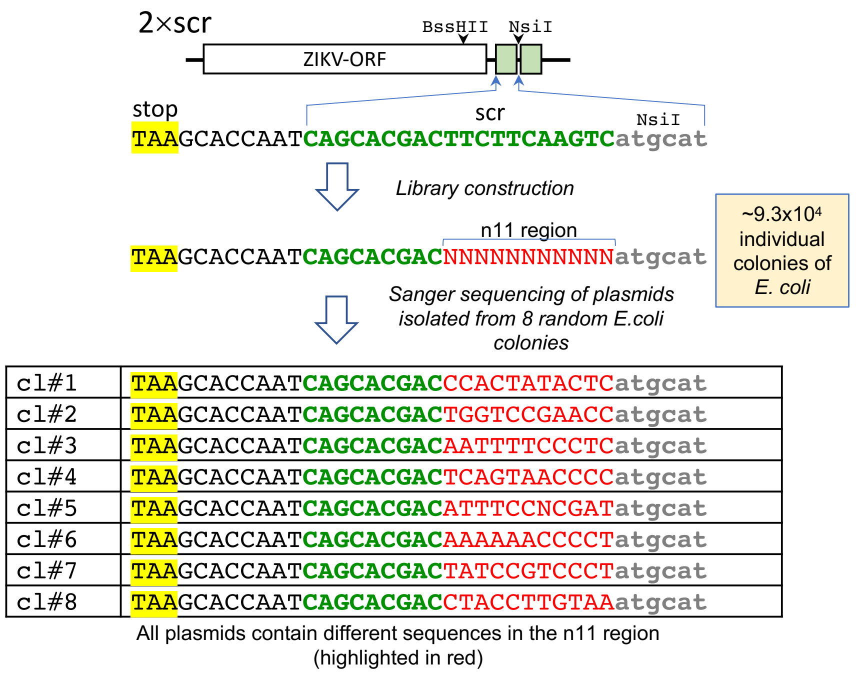

Figure S8. Sequencing of the 3’NCR of ZIKV-lib/n11 plasmids isolated from 8 randomly selected *E. coli* colonies. The n11 regions of the plasmids are highlighted in red.

Supplementary Table S1. Infectious titers and stability of 2×141(T) in the testis, epididymis, vas deferens, seminal vesicle/prostate and brain of AG129 mice at 17 dpi.

Mouse ID	Testis			Epididymis			Vas deferens		
	Titer [a]	Stability	Escape mutation(s)[c]	Titer [b]	Stability	Escape mutation(s)[c]	Titer [b]	Stability	Escape mutation(s)[c]
#1	5.38	Stb	-	3.40	Mut*	1) Δ (7-73)<br>2) Δ (17-135)	0.70	No PCR	-
#2	7.33	Stb	-	4.88	Stb	-	1.18	No PCR	-
#3	7.16	Stb	-	4.19	Mut**	1) Δ (21-135)<br>2) Δ (24-146)	2.45	Mut**	1) Δ (21-135)<br>2) Δ (24-146)
#4	7.18	Stb	-	3.19	Mut***	1) C_{19}→T + Δ (32-72)	1.94	Mut***	1) C_{19}→T + Δ (32-72)
#5	7.57	Stb	-	4.02	Stb	-	4.24	stb	-

Mouse ID	Seminal vesicle/Prostate			Brain		
	Titer [a]	Stability	Escape mutation(s)[c]	Titer [a]	Stability	Escape mutation(s)[c]
#1	1.70	No PCR	-	7.81	Stb	-
#2	1.70	No PCR	-	5.12	Stb	-
#3	2.83	Mut**	1) Δ (21-135)<br>2) Δ (24-146)	6.99	Stb	-
#4	1.70	No PCR	-	4.59	Stb	-
#5	1.70	No PCR	-	5.26	Stb	-

a – titer is expressed as Log_{10}(pfu/g); limit of virus detection is 1.70 Log_{10}(pfu/g)
b – titer is expressed as Log_{10}(pfu/mouse); limit of virus detection is 0.70 Log_{10}(pfu/mouse)
c – positions of the deleted or mutated nucleotides in the 3’NCR of 2×141(T) are indicated
Stb – stable (no mutations were found in the miRNA targeted region)
Mut – escape methanation(s) were found in the miRNA targeted region
No PCR – RT-PCR analysis was not reformed on this samples due to low virus titer

* - Deletions in the 2×141(T) virus isolated from the epididymis of **mouse #1**

TAAGCACCAAT**ccatcttt**~~**accagacagtgtta**_**ATGCAT**_ACCAATCTTAAT**ccatctttaccagacagtgtta**TGTTGTC~~

- Deletions in the 2×141(T) virus isolated from the epididymis, vas deferens, seminal vesicle/prostate of **mouse #3

TAAGCACCAAT**ccatctttaccagac**~~**agtgtta**_**ATGCAT**_ACCAATCTTAAT**ccatctttaccagacagtgtta**TGTTGTC~~

***- Deletions in the 2×141(T) virus isolated from the epididymis and vas deferens of **mouse #4**

TAAGCACCAAT**ccatctttac(c_{19}→T)agacagtgtta**~~_**ATGCAT**_ACCAATCTTAAT**ccatctttaccagacagtgtta**TGTTGTC~~

Supplementary Table S2. List of primers used in the study.

Primer name	5’→ 3’ sequence
ZV-9574-F	TGTGGCTGCTGCGGAGGTCA
Library-R	ATTGGTATGCATNNNNNNNNNNNNGTCGTGCTGATTGGTGCTTACAGC
ZV-10044-F	GGGAGAACTACCTGGTCAATC
ZV-10722-R	GGTCTTTCCCAGCGTCAATATG
ZV-10239-F	CGCACCACCTGGGCTGAGA
ZV-10489R	ACAGGCAGCATGGCTTCTTC
