# Supplementary material for: Epididymal epithelium propels early sexual transmission of Zika virus in the absence of interferon signaling
Source: Nat Commun. 2021 Apr 29;12:2469. doi: 10.1038/s41467-021-22729-5 (PMC8084954; doi:10.1038/s41467-021-22729-5)
Supplement: Supplementary file 3 — Description of Additional Supplementary Files [file 41467_2021_22729_MOESM3_ESM.pdf]

Description of Additional Supplementary Files

Epididymal epithelium propels early sexual transmission of Zika virus in the absence of interferon signaling

Pletnev et al.

Supplementary Data 1 legend: Sequences, number of detected reads (# reads), and frequencies of barcodes detected in the inoculum (Virus-Inoculum), in the male serum at 1 dpi (Serum 1 dpi), epididymis, testis, vas deferens, SV/P, male brain, and ejaculate (FRS) at 10 dpi collected from mice in mating pair # 1 (see in Fig. 6).

Supplementary Software 1 legend: Swarm Program Manual

Introduction:

The primary purpose of SWARM program is to analyze deep sequence data and perform some other functions. The functions include basic nucleotide database maintenance, alignment, phylogenetic analysis, creation of various sequence profiles, re-coding viral genomes, and identification of oligonucleotides appropriate for development of microarrays and MAPREC test. It also performs some other functions and can operate with protein sequences, and reconstruct phylogeny based on sequences comparison.

Unlike many other programs, multiple sequences do not have to be aligned in order to be analyzed by this program. SWARM can work with either sequences in GenBank or FASTA formats, and can use FASTQ files generated by Illumina HiSeq or MiSeq.

SWARM is based on command-line interface and works in UNIX (Darwin) Terminal (or similar applications) of Mac OS X operating system. It displays prompt "->?   " after which you can issue commands by typing on the keyboard followed by RETURN or ENTER. SWARM is normally installed in /usr/local/bin directory. Help file and some other supporting files should be placed in ~/Library/Application support/swarm directory. 'install' UNIX executable batch file is available to facilitate installation of SWARM.

More than one command separated by semicolon can be issued at the same time. In this case they will be executed sequentially. This feature is helpful for analysis of large data sets that takes a long time.

A batch job (script) with multiple commands can be executed. To do this create a plain text file (.txt, not .doc or .rtf) with a list of SWARM commands, save it, and then use its name as an argument to BATCH command. Alternaively, to launch batch process, you can issue command (in UNIX):

swarm job_batch.txt

In this case SWARM will quit after processing the batch job. See more in the section describing 'batch' command.

SWARM recognizes several options given from command line or in batch scripts:

swarm -c <command_name> <arguments...>

    will run a single swarm command and quit

swarm -s <command_name> <arguments...>

'map' command performed in batch mode. It launches another 'swarm' process.

swarm -d <directory_name>

is used to specify the directory name where to run 'slave' process

The list of all available commands can be retrieved through 'help' command.

A more detailed description of SWARM commands can be obtained by using

man command_name

For 'help' command to work, a text version of 'swarm_manual.txt' file must be present in ~/Library/Application Support/swarm/ folder.

SWARM results can be saved to files that can later opened by respective helper applications, such as MS Excel, MacVector, TextEdit, MS Word, FigTree, etc. They must be installed on the computer. Also, to use 'align' command please install 'mafft' application. It can be downloaded from:

http://mafft.cbrc.jp/alignment/software/macosx.html

Files containing results is done by issuing SAVE command on the keyboard, followed by the argument that tells the program what you want to save (see section about 'save' command). Saving (or printing) the dialogue (Terminal window) can be done by pulling SAVE command in the FILE menu, or PRINT command in the FILE menu.

The files containing the program results are given names that contain a common "basename" identifying the specific project you are currently working on. The basename is appended with extensions indicating the the type of file. For instance, if this is a text file, it is appended by either ".TXT" or ".DOC" extension so that it can be immediately opened by TextEdit or MS Word. If this is a phylogenetic tree, it is appended with ".TRE" so it can be opened by "FigTree" program. To change the "basename", change parameter 'output" by issuing:

output=basename

command, where "basename" is the word of your choice.

Similar to UNIX, SWARM uses the 'working directory' and 'home directory' concepts. You can use either complete UNIX file path, or just a file name. In the latter case it will be assumed that the file is located in the directory from which SWARM was launched. Top change the default ('working') directory you can issue

cd new_directory_path

from inside SWARM. you can also use 'pwd' command to determine which directory you are currently in.

To obtain the list of available commands with short explanations type "help". Unilke UNIX, all interactions with the program are case-insensitive, so "help" or "heLP" has the same meaning. To quit the program you should type "QUIT" or "EXIT", or simply "Q". SWARM understands abbreviated commands as long as the abbreviations match only one command. If there are more than one command matching the abbreviation, the error message will appear. For instance, commands "save", "summary", "show", and "slide" all start with "s", so to distinguish between them you must type at least two letters of the command.

Some commands allow you to specify options (modifiers) that specify the mode of action. For instance, you can say "save tree", or "save matrix", etc. In this case 'tree' and "matrix" are options, and they can also be abbreviated to first few letters.

The program performance is modified by a number of parameters that can be changed. For instance, melting temperature of oligonucleotides (Tm, in °C) or NaCl concentration (in mM), etc. Parameters can be changed by issuing commands such as: NaCl=100. Equal sign separates parameter and the value you want to assign to it. The list of current parameters values can be obtained by "ls" or "parameters" commands. For boolian parameters that can only have values 0 or 1 you can also use words yes, no, true, and false. For instance "interactive=yes" will set the value to 1, and "interactive=FALSE" will set the value to 0. Most parameters are automatically saved to a local preferences file so that next time you use the program you do not need to set up all the values again. The "Swarm preferences" file is created in each directory (folder) where the program has been run, and initially this file contains default parameter settings.

Format of the source data

Nucleotide and amino acid sequence information can be in different formats, including single or multiple files with individual sequences.More than one sequence can be present in one file. Sequences can be added to the set by repeatedly importing from different files, or by importing from multiple files as one operation. To do this, a list of files to be read must be places in a special text file (e.g. SimpleText, or text export from MS Word), and character '@' added in front of the filename when issuing "open" command:

open @filename

In this case the program will sequentially read all the files listed in the "filename" file.

Data types

The main data type is called "Sequence". It the sequences were aligned or have the same length, then the data become suitable for treatment by some algorithms that require alignment. If the initial data is not aligned and was imported from Genbank or FASTA files, the matching alignment information can be added by "re-opening" (see command "reopen" below)

respective aligned sequences file in either FASTA format. The aligned sequences can be saved or displayed on the screen:

save alignment

show alignment

To save multiple sequence alignment to a FASTA file use command

save afa

Alignments can also be shown or saved in "dot" format:

save dot

show dot

save dot   35

show dot   22

In this case the sequence is given only for the first sequence, and the remaining sequences show only those characters that are different from the first sequence. All identical bases are shown with dots. When saving in dot format there is an optional argument, which specifies the lead sequence to be displayed in the top line. All other sequences will be compared to it. When no argument is given, the first sequence in the set will be used as the lead.

Even if the sequences were aligned, they can be saved as Genbank or FASTA file without gaps ('-' symbols):

save sequence

export

The information about the sequences that have been read into the program can be obtained by issuing "summary" command. The information is both displayed on the screen and saved to a file "XXX Summary". Alternatively, "list" command will print out all sequence names and their lengths. "list 22" command will show the detailed information about sequence number 22.

>exit, quit

Quits the program and saves preferences. You can also force-quit by CTRL-C, but in this case the preferences will not be saved. Make sure you save/export your results before quitting, because it will give no warning that you have unsaved resulte.

>help

Displays the list of available commands with short explanations and available types of data with their legitimate names

>cd

Change working directory. Similar to UNIX command, changes the default folder in which files are opened. Please note that when you quit swarm you will remain in the UNIX directory where you've launched it.

>pwd

Print working directory. Similar to INIX command, shows the current directory path.

>rename

Renames multiple files according to the list specified in the argument file. The file can be in either CSV or tab delimited TXT format. It should contain two columns, one for the old names, and one for the new names.

Syntax:rename listfile.csv

>batch

To execute batch jobs. Swarm commands must be listed in a text file that is used as an argument. If one of the commands fails to execute, all subsequent commands in a block separated by an empty line will be ignored. After completion 'swarm' prompt will appear and the program will be ready for additional commands.

Syntax: batch 'batchfile.txt'

The name of batch file can be used as an argument of 'swarm' command when issued from UNIX prompt. In this case upon completion 'swarm' will quit.

Syntax: swarm 'batchfile.txt'

>man

Program manual. When issueds without argument, the command displays general introduction. Optional argument specifies the command that you want to learn about.

Syntax: man tree

>parameters, ls

Lists current parameter values and displays the results that have been calculated in this session and which are available to be exported / printed / saved.

>preferences

To read alternative preferences file. You can have several files that can be created from the saved "Swarm preferences" file by giving them unique names.

Syntax: preferences preference_filename.txt

>default

To reset all parameters to default values.

>list

Displays the list of sequences that have already been read and are available for immediate analysis. Optional argument specifies the subset of sequences to display. If only one sequence is available, the command will show detailed sequence information including available features in the sequence(genes, coding regions, etc.)

Syntax: list

List 258-290

>open

Opens and reads sequence data file. If the command has no argument the program will read the last file that was read (as specified in the "input" parameter that can be also changed by issuing "input=XXX" command). Alternatively, you can type the command with the optional argument specifying the file to be read: "open XXX". The program can read many different data types, including individual GenBank files and GenBank files containing multiple sequences (concatenated), FASTA files, multiple alignments in ClustalW, ClustalX, FASTA formats, as well as groups of files. After reading the data, the program will display the summary of all sequences it successfully imported.

To read more than one file the names should be listed a text file (e.g. created by TextEdit in MacOS, or saved as text (not RTF) from MS Word) so that each name was on a separate line. In MacOS such lists can be created by copying a group of names in Finder and then pasting them in TextEdit or MS Word. To open such lists type "open @XXX", where XXX is the name of the file

with the list of data files. Another way to open multiple files is to issue 'open' command several times, in which case new sequences will be added to previously read sequences.

'open' command can also be used to open other data files, such as deep sequence map files (mapindex and mapdata), binary SNP profiles, tree files in NEWICK or NEXUS format, and distance matrices in tab-delimited or comma-separated (CSV) formats.

If "Open" command is given starting with capital 'O' then the base-name of the input file is forced into the base-name of the output files and overwrites the previous base-name. Another way of changing the output files base-name is to change parameter 'output'.

>reopen

To add information from additional GenBank files or to read tree files, distance matrices, and saved search results. Aligned sequences read from multiple sequence alignments or FASTA files do not contain annotations and have only sequence name and the sequence itself. To add the missing info REOPEN command matches the accession numbers in the existing set (multiple alignments should be identified with names beginning with accession numbers of GenBank files from which the sequence was taken) with sequences in GenBank formatted file that is being reopened. It adds the data so that sequences can be properly identified and labeled. Reopening tree file will result in substituting the existing (if any) tree that was created from the sequences. Reopening matrix files will result in replacing the existing matrix (if any) with the new one.

>add

Add new sequences to the existing database. Similar to issuing multiple 'open' commands, but in this case duplicate sequences are not added. This command is useful for updating databases with new sequences.

>delete, remove

To remove specified sequences in the data set. Can also be used similar to 'drop' command. Syntax:

remove 1, 3, 7

remove 1-5, 5-13, 44

The command can be used with an option 'keyword' followed by the text to search for.

remove keyword "text-to-find"

To combine several keywords into logical OR expression, use '|' symbol:

remove keyword "L protein" | polymerase

Logical AND function can be achieved by repeating this command.

Similar command: 'drop' that allows to remove sequences based on such features as length, keywords, remove duplicates, etc.

>keep

To remove sequences other than those specified in the data set. The opposite of commands 'remove' and 'delete'. Can also be used as opposite of 'drop' command. Syntax:

keep 1, 3, 7

keep 1-5, 5-13, 44

The command can be used with an option 'keyword' followed by the text to search for.

keep keyword "text-to-find"

To combine several keywords into logical OR expression, use '|' symbol:

keep keyword "L protein" | polymerase

Logical AND function can be achieved by repeating this command.

>select

To remove sequences except those that are specified in a file by their LOCUS name, or by sequence number in the set. 'listfilename.txt' file contains only one column with no header. It can also be in 'csv' format.

select listfilename.txt

>clear

To erase the source data and/or results. If issued without an optional argument, it will erase all data. The argument ("alignment", "matrix", "oligonucleotides", "tree", etc.) should be given if you want to erase only some data.

>drop

To remove sequences meeting certain criteria. For instance, length less than N, or duplicate sequences. The command syntax:

'drop length < 7400'

'drop len > 7400'

will remove sequences with length less than, or greater than 7400, respectively.

drop duplicates

will remove duplicate sequences.

drop keyword "L protein"

will remove all sequences with the keyword found in features fields.

To combine several keywords into logical OR expression, use '|' symbol:

drop keyword "L protein" | polymerase

Logical AND function can be achieved by repeating this command.

>reduce

To reduce the size of sequence database by removing sequences that are closer than the specified value, or to reduce the database to a maximum number of sequences.

Syntax: 'reduce 5%'

'reduce 0.05'

'reduce to 100'

The value the distance between sequences, that is typically in the range between 0 and 1. In case 'normalize' parameter is FALSE, the distance matrix will contain values greater than 1 (actual number of mismatches). In this case the argument for the "reduce' command should indicate the minimum number of mutations. With argument 'to' the command will reduce the number of sequences to the specified value.

>filter

To remove sequences from FASTA files based on a user-provided keyword.

Syntax: filter "L protein"

>catenate

To merge (catenate) multiple text files into one file. The list of files should be presented in a text file and the result is saved in one file.

Syntax: catenate "filelist.txt"

>split

To split multiple GenBank format files into separate GenBank files named according to LOCUS field. Sequences could be already opened, in this case no argument needed. Alternatively, the command will split the file indicated by the argument.

Syntax: split [filename.gb]

>save

Saves the results or sequence information. In this case argument specifying what you want to save is required. It can be the data type that you want to export ("sequence", "genbank", "gb", "fasta" , "afa" (aligned fasta), "alignment", "dot", "matrix", "tree", "oligoprobes", "difference", "histogram", "preferences", "summary", "search", "snp", "profile", etc.). The list of available data types can be obtained by typing "ls" command. The name of the output file will be created form the basename appended with appropriate extension that is self-explanatory.

>show

Similar to the above "save" command, but displays the results on the screen. Requires argument. Some data types that require specialized graphics cannot be shown by this command, e.g. tree. TO visualize these types use save command, and open the resulting files in helper applications, such as FigTree.

>export

To export the sequences in GenBank format. Creates *.gb file (that can be opened in MacVector ot TextEdit) with all sequences catenated into one file. Optional parameter specifies the tree branch to be exported. Syntax:

export [FJ]

where FJ is the designation of internal node on the phylogenetic tree

>fasta

To export the sequences in FASTA format. Creates *.fas file (openable in MS Word, respectively) with all sequences catenated into one file. Optional parameter specifies the tree branch to be exported. Syntax:

fasta [FJ]

where FJ is the designation of internal node on the phylogenetic tree. The command is identical to save fasta command.

>definitions

To import definitions from a tabulated text Summary file that was created by "Summary" command in Swarm. The command is useful for adding / changing sequence attributes and information in case it was obtained from a multiple alignment or FASTA file. Sequence identification and matching is performed either by sequence length (in this case the number of sequences and their order must match) or by accession number (which is the sequence label in the multiple alignment file or the first word in the FASTA tag). The following columns can be changed in the Summary file:

Locus

Accession

Definition

Organism

Genotype

Serotype

Strain

Isolate

Note

Gene

Product

An additional column with "Label" identifier can be manually created (using Excel) to specify custom names of sequences to be used for printout of results.

>import

To import additional definitions from a tabulated text file (e.g. created in MS Excel and saved in tab-delimited text format). The additional definitions are added to the "SOURCE" field. First line of the first column of the file should contain the word "Accession" and first line of the second column should contain the keyword of the definitions field you want to add. For instance, if you add the "isolate" field it should be "   /isolate=", like in GenBank file. Please note that the number of spaces at the beginning of this field should exactly match the number in a real GenBank file, therefore you may want to copy-paste from a sample GenBank file. Starting from the second line of the tabulated file the two columns contain accession numbers and the new definition that you want to add to each sequence. Syntax:

import "filename"

>label

To provide alternative labels to be used in tree and multiple alignment printouts. The new names are imported from a text tab-delimited file in which the first column is accession number or other unique sequence identifier, and the second column is matching new label. This command is convenient to help generate publication-quality trees. Syntax:

label filename

If this command is issues without 'filename' argument, the label is created from select identifiers present in the original GenBank file, such as accession number, strain, gene, etc. The program will prompt to provide the sequence of fields that you want to include in the label. "definitions" command can be used for similar purposes.

>whois, whatis

To idenitfy members of a branch on the tree. Syntax:

whois B

whois AJ

>search

To search for oligonucleotides (for microarray design) that match sequences and group of sequences, and that can be used to differentiate them from the rest of sequences in the set. Can be used for both aligned and not-aligned sequences. If phylogenetic tree has not been previously calculated for this set, it will be done along with tree optimization. The command is controlled by 'searchmode' parameter which can have two values 0 (FALSE) or 1 (TRUE). If FALSE, then algorithm based on nucleic acid screening is used, if TRUE, then algorithm based on

protein screening (codon degeneracy) is used. Use of the latter algorithm could be done by either opening protein sequences file, and then adding respective coding sequences, or by opening nucleic acid sequences that contain markup of coding regions (CDS or mature peptide features in Genbank format). The protein sequence is then generated automatically.

Algorithm based on nucleic acid screening:

The algorithm will consider not only perfect matches, but also partially mismatched oligonucleotides. Currently maximum of one mismatch is allowed per oligoprobe. In addition, the margin of discrimination is also considered. This means that oligoprobes matching the desired set of sequences are analyzed to determine whether they are too close to homologous stretches in other sequences.

Algorithm based on protein screening:

The search command can also be used to screen protein sequences for conserved motifs, and also to create oligoprobes matching the conserved peptide motifs. If the command is issued for a set of protein sequences, it will ask whether you want to add a matching set of nucleic acid sequences. If not, it will simply work similar to the way it does in the previous algorithm. If yes, then after the conserved motifs consisting of low-degeneracy codons (amino acids coded by one or few codons) will be displayed at nucleic acid level.

The results are saved as follows. "SAVE oligonucleotides" saves the "XXX Search Map.doc" file showing the location of appropriate oligos. If the argument "oligonucleotides" starts with capital "O", then all results, including composite nodes will be printed out. Lowercase "o" will force to print out only clusters matching the tree topology. The file contains both individual sequences and groups named in the order of their creation by the cluster algorithm (as in phylogenetic tree reconstruction). The first ancestral sequence is named A, then B, and so on to Z, AA, AB, ... to ZZ, AAA, AAB, and so on. After all internal nodes of the phylogenetic tree are displayed, the so-called "composite nodes" are shown, only in case there are oligonucleotides that identify these "composite nodes". They are combinations of real and ancestral sequences (internal nodes on the tree) that cannot be placed in proximity to each other on a tree, but nevertheless share common oligonucleotides. This is helpful for finding oligonucleotides discriminating polyphyletic groups.

Search for oligonucleotides automatically generates distance matrix and cluster tree. It is highly recommended to print out the tree to facilitate interpretation of the "Search Map". Each ancestral (internal) node on the tree is marked with letters matching the names on the "Search Map". The nodes of the tree for which unique oligonucleotides were found are shown in uppercase letters, the rest of the nodes are shown in lowercase letters. Individual results mapped in this file can be saved individually with detailed sequence information and proposed consensus sequence by issuing "SAVE oligonucleotides 23" to save results for species number 23, or "SAVE oligonucleotides BZ" to save internal node BZ. Typing "SAVE oligonucleotides all"

will save all results. Data for each species and each ancestral node are saved in individual files named "XXX_23 Search" or "XXX_BZ Search".

Search for oligonucleotides is modified by some parameters. First, parameters "Tm" and "NaCl" determine the length of oligonucleotides that the program considers. Second, "tolerance" parameter specifies how many exceptions is allowed (mismatched sequences per sequences cluster or tree branch). This limit is applicable only to big branches with the number of sequences exceeding 'ctolerance' parameter value.

The results of "search" command can be saved for future analysis and use by using "save search" command. The binary file "basename.SEARCHDATA" with search results will be created and can be later reopened. To do this first read respective sequences, create (or reopen) a tree, and then:

reopen somename.SEARCHDATA

The data will be read into the program, and individual results for each branch of the tree can be saved by using :

save oligo XYZ

command

>iupac

To replace IUPAC symbols with 'A'.

>lefttrim

To remove nucleotides from the 5'-end. Sequences must be aligned first.

Syntax: lefttrim <number_of_nucleotides>

>leftrestore

To copy missing nucleotides from the 5'-end of the reference sequence present in the same aligned set of sequences.

Syntax: leftrestore <number_of_nucleotides> <reference>

>polyatrim

To remove poly-A tract at the 3'-end.

>verifycds

To verify CDS information. Checks is coding sequences contain stop codons or are not multiples of 3. Interactive mode.

>shred

To split very long sequences into several genbank files. This command is needed because of the limitation on the size of sequence files used as reference for mapping deep sequence data (currently 32,000).

>markup

This command is similar to "fetch" but instead of fetching parts of sequences based on annotations contained in lead sequence, it adds similar annotations to the rest of the sequences. To use this command you need both Genbank file with annotated sequences, and their alignment created by Clustal program. First open sequences, then "reopen" alignment, and then "markup". Syntax:

markup lead_sequence

lead_sequence is the sequence containing annotations you want to use. It can be in the form of sequence number in the existing set, or a path to the reference sequence.

You will be prompted to indicate what feature you want to markup; there is an option to markup all of them.

>fetch

This command is similar to "extract" but it requires only one sequence to have all the annotations (features) describing location of coding sequences, genes, etc. To use it you need both Genbank file with annotated sequences and their alignment created by Clustal or mafft programs. First open sequences, then "reopen" alignment, and then "fetch". Syntax:

fetch leadsequence

leadsequence is the sequence containing annotations you want to use.

You will be prompted to indicate what feature you want to fetch; there is an option to fetch them all. Each feature will be extracted and saved in a separate ***.gb file (Genbank format) and ***.afa (aligned FASTA file), where *** is gene name.

>sfetch

To display individual sequence reads.

>extract

To extract parts of sequences specified in FEATURES fields of GenBank files. In the current version the recognizable features include CDS (coding sequence), mat_peptide (mature peptide), and GENE (gen). The name after the first colon specifies the sub-title in the feature, e.g. product, gene, protein_id, note. Either or both can be left blank; in this case all implemented sub-titles will be searched. If more than one matching feature is found, you will be given a choice. Please select the one you want to be extracted by typing the appropriate number. Command syntax:

extract CDS:product:polymerase

extract mature::nucleoprotein

extract ::"VP1 protein"

In the first case nucleoprotein coding sequences specified by "product" line will be extracted. In the second example all fields (product, gene, protein_id, note) will be searched for match with "nucleoprotein" keyword". In the third example all Features fields will be searched.

>chop

To split a short nucleotide sequence entered from the keyboard into oligonucleotides of defined Tm. Used to design oligonucleotide microarrays. Syntax:

chop ACTCGCTATTCGCTATGGCT

The output on the screen includes the length and G:C contents of each oligonucleotide. They can be copy-pasted to another application for further use.

>align, mafft

To align multiple nucleotide sequences using 'mafft' helper program. It must be properly installed before execution of this command, see website http://mafft.cbrc.jp/alignment/software/. The command has two options controlled by 'cds_alignment' parapeter. If FALSE, alignment is performed based on nucleotide or amino acid sequences of the sequence set previously opened by 'open' command. If TRUE, alignment of 5'-ends and 3'-ends is done by nucleotide sequences, while the ORFs are aligned based on their translation into amino acid sequences. Only one ORF is allowed, and this version of the command is suitable for Enteroviruses, Flaviviruses, or other groups with a single ORF. The

source file should be in Genbank format, and have CDS information. If no CDS information is available, the longest ORF will be used. All CDS and mat_peptide features are quality controlled to trim stop codons, and ensure that their length is a multiple of 3.

Syntax: open filename.gb; mal

open filename.gb; mafft

The aligned sequences set can be saved by 'save afa' command. Intermediate files (aligned 5'-ends, 3'-ends, and amino acid sequences) are automatically removed if 'cleanup' parameter is set at TRUE.

>differences

To print out differences between sequences two or more aligned sequences.

Syntax:difference [sequence_number]

Optional argument sequence_number indicates the lead sequence with which to compare others. If no argument is given, then the first sequence will be used.

The result includes all differing positions with their coding information (gene, position within the gene, relevant amino acid substitutions), as well as the contribution of individual mutations to the difference between the lead sequence and the rest. If lead is not specified, then contribution is computed from comparison of all sequences among themselves.

>diversity

To create diversity profile of a sequence. The results are saved to Excel file.

>simplot

To generate simple. Sequences must be aligned. If sequences contain markup, each feature is compared before scanning with a sliding window.

Syntax: simplot [a-b] [x-y] to analyze sequence a through b, and to exclude from comparisons sequences x through z.

>scantree

To scan multiple alignment with a sliding window ("SlideWindow") to generate distance matrices and determine tree topology. Can be used with both aligned and non-aligned sequences. In the latter case a "lead" sequence must be specified as a reference. Window movement is done by half-length (50 bases if the SlideWindow valie is 100). Can only be done

with aligned sequences. The results are saved in "XXX Treescan matrices.xls" file that can be used to open matrices in MS Excel, and "XXX Treescan trees.tre" file that contains multiple tree information in Nexus format that can be opened in TreeView program. Saving matrices is optional, and can be disabled in the dialogue that is self-explanatory. Optional parameter "optimize" is used to optimize topology of the trees. It will significantly increase the time needed for the scan. Syntax:

scantree

scantee optimize

>toposcan

To scan multiple alignment with a sliding window ("SlideWindow"), to reveal some topological properties of their phylogeny. Can only be done with aligned sequences. The results are saved in "XXX Topology Scan.xls" file that can be used to graphically plot the profiles in MS Excel. For each position in the alignment, the file contains a number of calculated values. Diversity2 is the same value as "Base diversity" in the "profile" command. The "mutation #" is the minimum number of mutations that may explain the existing base variability at a position. For instance, if there is only C and T at a particular site, the number is 1, if a protein site contains A, C, G, and H, then the minimum number of mutations is 3. "tree length" is the number of mutations at this site that can be inferred from the overall topology of the tree, including back mutations and parallel mutations. In ideal (additive data) situation these numbers should be identical, their difference implies that the tree topology is a compromise between conflicting information coming from different parts of the sequence. The level of this "conflict" or "non-additivity" is expressed in %% in the next column. For instance, if the minimum number of mutations is 1 and in the reconstructed tree there is 5 mutations at this site, the "non-additivity factor" is 400%. Differing quartets are calculated by comparing two sliding windows shifted by one base relative to each other, and determining topology of all possible sub-sets of four sequences. The quartets whose topology differs in adjacent windows are counted, and serve the measure of potential disturbances of topology of the entire set, that can be for instance a result of recombination. The topology is calculated from a distance matrix based on the theorem of four nodes. The theorem says that for any subset of four nodes of an additive tree pairs of distances between the nodes satisfy the following rule:

$D_{a,b} + D_{c,d} = D_{a,c} + D_{b,d} > D_{a,d} + D_{b,c}$

Therefore one can unambiguously determine topology of any and each sub-set of four nodes, and there is only one way of putting them together in a joint tree. The number of "quartets" that have different topology can be a measure of disturbances of that are due to mutations and genetic recombination. This value is also calculated separately for all quartets that include individual nodes. These plots are helpful to identify potential crossover points involving individual sequences.

>relatedness

To look for relatedness among sequences. Can be used with both aligned and non-aligned sequences. In the latter case a "lead" sequence must be specified as a reference. There are two modes: whole sequence and scan with a sliding window. In first case the printout consists of two columns, each line for each sequence in the alignment. In the second case each column corresponds to a sequence, and each line show relatedness within a sliding window. The window width is determined by a parameter "RelatWindow". The command can only be performed with aligned sequences. The results are saved in "XXX Relatedness.xls". XXX consists of "basename" and the number of sequence in the array. The plot that can be used to graphically present the profiles in MS Excel. The plot is useful in search for potential recombinants. Syntax:

relatedness 25-27

relatedness scan 25-27

to analyze sequences 25 through 27. Each sequence will be analyzes separately and saved in individual file.

>profile

To calculate diversity profile of multiple sequence alignment. Can only be done with aligned sequences. The results are saved in "XXX Diversity Profile.xls" file that can be used to graphically plot the profile in MS Excel. The program splits the sequences into oligonucleotides of the preset Tm at the specified NaCl, and then determines how much base diversity and oligonucleotide diversity there is in each position of the multiple alignment. "Oligo Diversity" is the number of different oligonucleotides in a particular column in the multiple alignment. "Base Diversity" is a sort of measure of "entropy" at a particular position in the alignment: it is equal to Zero if the position is completely conserved, and 1.0 if it is totally random. The formula is:

(N2 – SUM(Mi,j)^2) / (N^2 /(1-m))

where N is the number of sequences in the array, Mi is the number of sequences with character 'i' at the position, and m is the number of characters in the alphabet (4 for DNA and 20 for protein).

"Base Diversity3" is calculated as a fraction of all possible pairwise comparisons at a particular base location. The formula is:

SUMi,j(Mi,j) / N^2

Where Mi,j is mismatch between sequences "i" an "j", and N is the total number of sequences. Mismatch is calculated based on Watson-Crick rules.

Syntax:

profile [AC-AE]

Optional arguments of this command refer to internal nodes on the tree (branches) as specified in PhylipTree output viewable in TreeView. In the case only sequences belonging to this branch are analyzed. Profile for each branch is saved into individual file.

>similarity

To calculate similarity matrix (and to make a respective cluster dendrogram) of non-aligned sequences by using a fast algorithm. It generates all available short oligonucleotides or oligopeptides to screen the entire sequences and to create a "fingerprint" that is used to assess differences between sequences.

This algorithm of distance matrix calculation is used also in 'matrix' command if the "oligomatrix" parameter value is 1.

First, the number of short oligonucleotides ("k-mers") that are unique to two sequences is determined. Then one sequence is randomized and the number of non-common k-mers is counted.

The algorithm uses the following empirical formula:

raw distance d = noncommonK-mers / randomnoncommonK-mers;

This measure of distance between sequences is non-linear with respect to the distance based on direct comparison of aligned sequences.

corrected (linearized) distance is

D = 10 ^ -(2 * SQRT (ABS (Log10 (d))))

The resulting parameter is reasonably linear, but reaches saturation for distances above ~25% nucleotide differences, and therefore should be used for reasonably related sequences only.

>bestmatch

To find the best match for sequences. The query sequences must be opened first with 'open' command. The function uses k-mer fingerprinting method. The results are displayed on the screen and saved to MS Excel file. Optional second argument [sequence_number] shows one sequence for which several top matches are displayed. The number of displayed results is determined by 'results' parameter. If this optional argument is omitted, the one top match for

all sequences will be shown. If 'reference_database' argument is omitted, the previous database will be used.

Syntax: bestmatch [sequence_number] [reference_database.gb]

>find

To find regular expressions, patterns, and consensuses in sequences. Degenerate symbols and variable length connecting stretches are allowed.

Syntax:find AGCC[GC]TT8-12AYYSG*CGC<AT>ATG

Square and angle brackets [ ] and < > enclose degenerate symbols. [ ] means including those that are inside, < > means excluding those that are inside. Range of numbers means a stretch of any symbols of respective length, and * means a sequence of any characters of unlimited length.

>translate

To translate a set of nucleic acid sequences and to print them out as multiple alignment with both amino acid and nucleotides on separate lines. The module requests to provide a list of unrelated sequences to identify and mark the positions in the sequence that consistently contain mutations, and therefore may be under selective pressure. Such positions are shown by asterisks and are highlighted in low-case letters.

>protein

To translate a set of nucleic acid sequences and to print them out as a GenBank file. 'CDS' or 'mature peptide' features need to be present. Alternatively, the program will give you a choice to create peptides from the longest open reading frame or from the beginning of each sequence. The function also checks validity of CDS and MATURE_PEPTIDE information, and offers to correct if the length is not a multiple of 3. It also trims stop codons at the end, so it is recommended to 'export' sequence file after this command to save these changes.

>orf

To display the longest open reading frame for each sequence. The sequence must be opened first.

>convert

To replace the set of nucleic acids with respective set of translated protein sequences. 'CDS' or 'mature peptide' features need to be present. Alternatively, the program will give you a choice to create peptides from the longest open reading frame or from the beginning of each sequence.

>composition

To calculate amino acid composition at each locus.

>mutate

To find mutagenic codons that will not give rise to the original sequence.

Syntax:mutate ATG (codon)

mutate A (amino acid)

Will print out degenerate codons in IUPAC code and the frequency of their use.

>codonprofile

To plot the number of synonymous codons coding each amino acid in alignment. Only those position in which a single amino acid constitutes more than a certain threshold of percent is treated, others are skipped. Parameter "codonprofilelimit" specifies the threshold. For instance, the default value 0.95 means that if no amino acid constitutes 95% or more in each column in the alignment, the position will be skipped.

>recode

To recode nucleotide sequence using different codons.

>restmac

To find restriction enzymes and appropriate modifications of PCR primers to be used in MAPREC assay.

Syntax:restmac [sequence number]

The optional argument specifies the sequence to analyze in case the nucleotide sequence data contains more than one sequence. The results are saved to MS word file. The information about restriction sites is contained within RESTMAC.DAT file that must be present in the current folder or ~/Library/Application Support/swarm/ folder.

>dmaprec

To prepare restriction enzymes database for MAPREC command. The source text file must contain two tab-delimited columns, one with name and another with recognition site in IUPAC format. Non-IUPAC characters are ignored. It is better to remove isochozomers from the source file before executing this command.

>slide

To split the sequence into a set of overlapping oligonucleotides. The size of oligonucleotides is determined by "Tm" and "NaCl parameters", and minimum length ("oligolength"). The overlap factor is controlled by "overlap" parameter. It the parameter is 2, the next oligo will start at half-length, if the parameter is 4, then it will be shifted by 1/4 of the length of the previous one. The command will lead to a dialogue that is self-explanatory. The results will be saved in "XXX Overlapping oligos.xls" file that can be opened in MS Excel. XXX stands for the basename that is used-defined. The file contains sequences, and all other necessary information about the oligonucleotides.

>fish

To determine the closest relative of 'lead' within the sequence database by using short fragments algorithm. Optional argument points to the lead or query sequence.

Syntax: fish 44

Where 44 is the number of sequence in a set.

>fishscan

To create a scan plot of closest relatives of 'lead' within the sequence database by using short fragments algorithm (fish).

Syntax: fishscan 44

Where 44 is the number of sequence in a set.

>matrix

To calculate distance matrix based on sequence data. If the sequences were aligned, i.e. have the same lengths, the matrix is calculated by counting nucleotide mismatches. In case the set of sequences was not aligned and they have different lengths, the matrix is calculated based on the number of matches of short sequence fragments from one sequence matching the other sequence (k-tuplet counting method). In case the matrix was calculated before, it is automatically created when you perform "search" command, or build phylogenetic tree.

There are several modes in which the matrix is calculated. The modes can be switched by the parameter matrixmode by a assigning a numeric value:

1 - simple mismatch calculation

2 - missense (coding) mutations only

3 - synonymous (silent) mutations only. The module asks to provide a list of sequences within the given set that are presumed to be unrelated. It is used to determine positions of the sequence that consistently contain mutations (more than 2 in the current implementation) and therefore are likely to be under selective pressure. These positions are disregarded and are not counted.

4 - shared synonymous mutations. This is used for analysis of multiple derivatives of the same strain, e.g. vaccine-derived viruses. Sequence number one is used as a reference to detect synonymous mutations, and then common synonymous mutations in each pair of sequences is calculated. The module asks to provide a list of sequences within the given set that are presumed to be unrelated. It is used to determine positions of the sequence that consistently contain mutations (more than 2 in the current implementation) and therefore are likely to be under selective pressure. These positions are disregarded and are not counted.

5 - the number of shared mutations that can be expected to be common in pairs of sequences simply by chance. The module asks to provide a list of sequences within the given set that are presumed to be unrelated. It is used to determine positions of the sequence that consistently contain mutations (more than 2 in the current implementation) and therefore are likely to be under selective pressure. These positions are disregarded and are not counted.

6 - the net number of shared synonymous mutations (same as option number 4 less option number 5). Provides the measure of common evolutionary pathway in a pair of sequences derived from common ancestor. The known common ancestor sequence is listed as number one in the analyzed set. The module asks to provide a list of sequences within the given set that are presumed to be unrelated. It is used to determine positions of the sequence that consistently contain mutations (more than 2 in the current implementation) and therefore are likely to be under selective pressure. These positions are disregarded and are not counted.

>tree

Depending on 'treemethod' parameter, builds tree by either cluster algorithm, or aggregate algorithm. If treemethod=0, cluster tree is built, alternatively if treemethod=1 aggregate tree is built. In the first case, depending on 'pearson' parameter, it will either assemble tree by pairing sequences with the minimal distance, or by pairing the sequences for which Pearson correlation coefficient between respective rows in distance matrix are minimal. In the second case tree is build by progressively adding more species to the location on the partial tree where they fit best. This method is slower but produces better trees. To minimize the possibility of getting

trapped into a local minimum, optimization using "hop" or "tear-off" algorithm can be performed after the tree is built. To enable this feature set 'treemode' parameter should be set to 1. Optional argument of the 'tree' command specifies the node of the final tree that will be placed on top of the tree (for presentation purposes). The command reconstructs branches lengths by either sequence reconstruction method, or from distance matrix. In case of distance matrix it can either reconstruct symmetric branches or asymmetric branches. This is controlled by 'branchsymmetry' parameter, either 0 or 1.

Syntax:

tree

tree 22

Where 22 is the lead sequence in which markup showing features is expected. This will prompt dialogue to identify the feature to be used for constructing tree instead of the entire sequence.

>optimize

Depending on 'optimmode' parameter, refines tree topology by either local quartet optimization (1), by global topological optimization ("hop" algorithm (2), or by two-step combination of the two (3). The algorithm attempts to reconfigure internal branches of the tree to minimize the number of discrepant quartets. It may take substantial time to process large data sets because the complexity of this algorithm is N4. Local algorithm rearranges only adjacent branches, while global tears-off one branch at a time, and finds the best location for each of them. The printout includes the results of assessment of topological quality of each node and of the entire tree. Intermediate tree structures and the progress of optimization are saved to respective log files. Optional argument specifies the individual internal node (branch) that needs to be optimized. If no argument is given, recursive optimization of all internal branches will be performed until minimum of discrepant quartets is reached. Syntax:

optimize

optimize BL

Intermediate trees generated in the course of optimization are saved in a separate file to be viewed in TreeView or TreeEdit programs, as well as the log of optimization process (MS Excel).

>move

To move a branch specified by the first argument to another location on the tree that is immediately below the branch specified by second argument. New branch lengths will be calculated, and tree topology will be re-analyzed. Syntax:

move AC BF

>hop

To optimize tree topology by \"tear-off\" or \"hop\" algorithm. Tree must be calculated or opened first.

>reconstruct

To reconstruct ancestral sequences and to refine tree branches in case the source sequence set is aligned. If the initial set of sequences is not aligned, distance matrix is used to calculate lengths of individual tree branches.

>insideout

To turn the tree inside out so that a particular tree node will be placed on the top of the tree (first line in the printout). Syntax:

insideout AH

insideout 23

>sort

Sorts the sequences based on phylogenetic tree by placing closely related species together.

>topology

To create phylogenetic tree using a new topological algorithm. This module is under construction. Use of this command may lead to unpredictable results

>pentanode

To analyze data using a pentanode algorithm. Under construction.

>control

To validate pairs of FASTQ files and to remove adapters and barcodes. The command performs quality control of sequences and creates MS Excel file with the distribution of PHRED scores and ACGT content.

>fastq

To validate pairs of FASTQ files and to remove adapters and barcodes. One '*_clearfastq.txt' file and log file are created. The command performs quality control of sequences and creates MS Excel file with the distribution of PHRED scores and ACGT content. The command can also tril sequence reads to remove the beginning and the end. Parameter 'miseq' must be TRUE if FASTQ files contain sequences of unequal length. Parameters 'fqstart' and 'fqend' determine the beginning and the end of the trimmed sequence reads. Parameter ‘removeadapters’ specify if the adapters must be removed.

>shuffle

To randomize 'clearfastq' files several times and select a specified number of paired sequence reads.

Syntax:shuffle 'mapbasename'

The module will request the number of sequence reads to select, and the number of replicates. The output is a series of randomized 'clearfastq' files with appropriate suffixes.

>map

To map deep sequence reads to reference amplicons sequences.

Syntax: map 'mapbasename'

map

Without argument it will use the last 'mapbasename' used. Filename of FASTQ or CLEARFASTQ file could also be used as the argument. In this cases 'mapbasename' will be determined automatically.

Where 'mapbasename' is the common part of the name of two paired end deep sequence FASTQ files. For instance, for 'Sample_1_R1.fastq' and 'Sample_1_R2.fastq' files the mapbasename is 'Sample_1'. The command is dependent of ampliconfilename. Output is saves into two large mapped data files (INDEX file and DATA file), and also match histogram (if matchhistogramprint parameter is TRUE)

>bias

To filter artifactual mutations based on polarity bias and distribution bias (Shannon entropy calculation). The results are saved to 'mapbasename_snpp.binary' file. SNP profile in tabular format can be saved by 'save snp' command, and stacks of sequence reads matching a mutation or range of mutations san be saved by 'save stack' command. Distribution of mutations along sequence reads and entropy calculation can be saved by 'save distribution' command.

Relevant Boolean parameters for 'bias' command include:

polaritybiasfiler Whether to filter SNP profile for polarity bias

distributionbiasfiler Whether to filter SNP profile for distribution bias

Relevant parameters for 'save snp' command include:

subtractbackground Whether to subtract background from SNP profile

mutbackgroundfile=FILENAME

consensusprint Whether to print corrected consensus sequence

muthistnormalize Whether to normalize mutations distribution histogram

savebackground Whether to save mutations background on file

entropy_cutoff Threshold of entropy to invalidate mutations (default=0.65)

>report

To print out deep-sequence mapping report.

>libsize

To printout statistics of library size distribution.

>classify

To classify individual sequence reads into discrete sub-populatiotns.

Syntax:classify [mapbasename]

Without argument it will use the last 'mapbasename' used.

>snp

To calculate SNP profile from previously mapped deep sequence reads.

Syntax: snp [mapbasename]

Without argument it will use the last 'mapbasename' used.

The command is dependent on the 'ampliconfilename'. It saves results into 'mapbasename_raw_snpp.binary' file. SNP profile in tabular format can be saved by 'save snp' command, and stacks of sequence reads matching a mutation or range of mutations san be saved by 'save stack' command.

Relevant Boolean parameters for 'snp' command include:

snp_mutrate_threshold Threshold of mutations rate in a sequence read acceptable for calculating SNP profiles

ref_summary Whether to save SNP relative to a reference sequence specified in 'referencefile' string parameter

>assemble

To assemble deep sequence reads into a contig based on multiple alignment of reference sequences. Deep sequence reads must be first mapped on multiple references file using 'map' command. Aligned multiple references fasta file must be present (specified by parameter 'alignmentfilename').

Syntax: assemble 'mapbasename'

Assemble

The command results in a GenBank file of consensus sequence, a simpolot printout of its comparison to the reference sequence set, and scan of modalities along the genome.

>profmatrix

To calculate distance matrix based on SNP profiles in tabular (CSV) profiles saved by 'save snp' command, or files generated by HIVE. File names should be listed in a text file. Parameter 'mincoverage' specifies the minimum valid sequencing coverage.

Syntax: profmatrix 'List.txt'

>profsummary

To save to CSV file a summary of all mutations in all SNP profiles that exceed a given threshold, and provides information about amino acid substitutions in genes according to GenBank markup. SNP profiles in tabular (CSV) profiles saved by 'save snp' command, or files generated by HIVE are used as a source (file names should be listed in a text file). Prints out contribution of each nucleotide to the diversity of profiles. Parameter 'mincoverage' specifies the minimum valid sequencing coverage. Positions below the threshold are disregarded and shown as negative numbers.

Syntax: profsummary 'list.txt'

>profdifference

To compare two sets of SNP profiles and identify nucleotides responsible for the difference between them. Unlike 'profsummary' command prints out sum of all mutations in each position. Parameter 'mincoverage' specifies the minimum valid sequencing coverage. Positions below the threshold are disregarded and shown as negative numbers.

Syntax: profdifference 'list1.txt' 'list2.txt'

>findumi

To find unique molecular identifiers immediately following an anchor sequence. Uses paired .fastq files as input. Saves the list (profile) of all UMI's found in .fastq files.

Parameters affecting the function:

anchoroligo = the oligonucleotide sequence immediately preceding the UMI

umilength = the length of the UMI

>umidistance

To calculate relatedness of UMI profiles from individual CSV files specified in the 'list' file. The argument is the text file with names of csv files with UNI profiles generated by 'findumi' command

Usage: umidistance list.txt

>umisummary

To make a summary of several UMI profiles. The argument is a text file with a list of UMI profiles generated by findumi command

>umiprofile

To calculate UMI profile based on *_I1.fastq file

>disequilibrium

To determine whether mutations are on the same molecule. Calculates the linkage disequilibrium for two mutations. Deep sequence reads mapped on a template must be first opened using "open' command. The function works in self-explanatory dialogue mode.

>mix

To mix (spike) deep sequence reads from two sources in a preset proportion.

>pair

To match paired end reads.

>usort

Obsolete function. To sort multiplexed ultra-deep sequence file and remove barcode tags.

>ultradeep

Obsolete command. To analyze ultradeep sequence data (massively parallel sequencing)

Syntax:ultradeep filename.fastq

A file containing template sequence and optional amplicon sequences must be opened prior to issuing this command

Can read a variety of HTS data format, with quality information in the same or different file. Saves a number of out put files, including a re-formatted data file suitable for re-analysis at a later time.

If only one sequence is given in the template file (GenBank format), it is presumed to be a compete sequence that is a single amplicon. If more than one sequence, then the first one is a template, and subsequent files are amplicons. Forward and reverse primers must be specified in the features section of the GB file, and also a key word "amplicon" must be present in "misc_feature" field. Regions specified as primer binding sites (primer_bind) will not be analyzed.

    primer_bind   1..35

/note=FORWARD PRIMER

misc_feature 36..2351

/note=AMPLICON

primer_bind 2352..2386

/note=REVERSE PRIMER

The function produced Excel files with mutations, as well as a new consensus sequence calculated based on the HTS data. Mutation frequencies tables are also saved.

>pdb

To extract protein sequence information from PDB files with 3-d structures.

Syntax:pdb filename.pdb

Creates respective filename.txt file

>atr2tree

Obsolete command. To convert *.atr and *.gtr files (alternative tree structure record produced by other programs) to either Phylip or Nexus format, depending on the value of "treeformat" parameter. The file specified in the argument to this command is used as the source, and the output is written into a file with respective name extension. Syntax:

atr2tree testdata.gtr

>marsh

To process files generated by microchip scanner for MARSH microchips (overlapping oligonucleotides for mutational screening). The data file should be in tab-delimited text format, and contain information about two hybridizations with homogeneous reference sample (microarrays 1 and 2), and two hybridizations with test samples (microarrays 3 and 4). Information about oligonucleotides must be in a separate tab-delimited text file (produced by editing files generated by "slide" command of Swarm). Reference oligoprobe information (e.g. probe matching the region corresponding to PCR primers or other conserved region with no sequence heterogeneity) should be added at the end of this file, and must be repeated as many times as the reference oligoprobe is printed on the microchip at the end of each sub-array. The program will ask for the name of file containing the oligoprobes list. The results including

average relative intensity (normalized by the reference oligoprobe or average intensity of the entire microchip, depending on marshnormalization parameter), standard deviation, and ratio of Reference 1 to each Sample will be saved in the file the name of which will be taken from the "Experiment" field in the source data file. The data is being filtered to remove outliers, that are more than 2 sigmas off the average value for each oligoprobe. The name of previously used file and the number of arrays / sub-arrays on each slide are saved as default values, and can be retrieved by responding with ENTER. Syntax:

marsh scanfile.txt

marsh

>flu1

To find oligoprobes distinguishing one sequence from all other in the database. Optional argument of this function indicates the lead (query) sequence. If no argument, it assumes that the first sequence is the lead. Saves results in appropriate output file in MS Word format.

Syntax: flu1 15

>a1

To perform analysis of microarray data from Measles genotyping chips. Each specific probe has a negative control, and the ratios of specific to the non-specific probes is calculated and saved in a file. Then comparison with the existing database of previous data for different genotypes is done and the resulting distance matrix (calculated by Pierce correlation) and the tree showing relatedness are saved. The name of previously used file and the number of arrays / sub-arrays on each slide are saved as default values, and can be retrieved by responding with ENTER. Syntax:

a1 scanfile.txt

a1

>a2

Obsolete function to be removed. To process database of Measles genotyping microarray results.

>ab1

Obsolete function to be removed. To open AB1 files and save in FASTA format

>hash

Obsolete function to be removed. To generate hash index of sequences for fast search.

>hsimplot

Obsolete function. To calculate simplot from deep sequencing data using 12-mer seed hash.

>htest1

Obsolete function. To find 12-mer seeds using hash.

>htest2

To match deep sequence reads with hash.

>pca

Principal component analysis (unfinished function).

>test

Area under construction. This module is used for new development.
