# Supplementary material for: Epididymal epithelium propels early sexual transmission of Zika virus in the absence of interferon signaling
Source: Nat Commun. 2021 Apr 29;12:2469. doi: 10.1038/s41467-021-22729-5 (PMC8084954; doi:10.1038/s41467-021-22729-5)
Supplement: Supplementary file 6 — Reporting Summary [file 41467_2021_22729_MOESM6_ESM.pdf]

nature research

Corresponding author(s): Konstantin Tsetsarkin

Last updated by author(s): 03/18/2021

Reporting Summary

Nature Research wishes to improve the reproducibility of the work that we publish. This form provides structure for consistency and transparency in reporting. For further information on Nature Research policies, see our Editorial Policies and the Editorial Policy Checklist.

Statistics

For all statistical analyses, confirm that the following items are present in the figure legend, table legend, main text, or Methods section.

n/a	Confirmed	
☐	☒	The exact sample size (n) for each experimental group/condition, given as a discrete number and unit of measurement
☐	☒	A statement on whether measurements were taken from distinct samples or whether the same sample was measured repeatedly
☐	☒	The statistical test(s) used AND whether they are one- or two-sided<br>*Only common tests should be described solely by name; describe more complex techniques in the Methods section.*
☒	☐	A description of all covariates tested
☐	☒	A description of any assumptions or corrections, such as tests of normality and adjustment for multiple comparisons
☐	☒	A full description of the statistical parameters including central tendency (e.g. means) or other basic estimates (e.g. regression coefficient) AND variation (e.g. standard deviation) or associated estimates of uncertainty (e.g. confidence intervals)
☐	☒	For null hypothesis testing, the test statistic (e.g. F, t, r) with confidence intervals, effect sizes, degrees of freedom and P value noted<br>*Give P values as exact values whenever suitable.*
☒	☐	For Bayesian analysis, information on the choice of priors and Markov chain Monte Carlo settings
☒	☐	For hierarchical and complex designs, identification of the appropriate level for tests and full reporting of outcomes
☒	☐	Estimates of effect sizes (e.g. Cohen's d, Pearson's r), indicating how they were calculated

Our web collection on statistics for biologists contains articles on many of the points above.

Software and code

Policy information about availability of computer code

Data collection	Software(s) was not used for data collection
Data analysis	Statistical analysis was performed using Graph Pad Prism 8 for Mac OS X. Digital slides were analyzed using ImageScope and Aperio Spectrum eSlide Manager software (Version 12.4). Bioinformatic software (SWARM) used for data analysis in the current study is provided in Supplementary software 1.

For manuscripts utilizing custom algorithms or software that are central to the research but not yet described in published literature, software must be made available to editors and reviewers. We strongly encourage code deposition in a community repository (e.g. GitHub). See the Nature Research guidelines for submitting code & software for further information.

Data

Policy information about availability of data

All manuscripts must include a data availability statement. This statement should provide the following information, where applicable:

- Accession codes, unique identifiers, or web links for publicly available datasets
- A list of figures that have associated raw data
- A description of any restrictions on data availability

The authors declare that all data supporting the findings of this study are available within the paper and its Supplementary Information. Full-length sequences for all viruses and for all cDNA infectious clones used in this study can be deduced from the information provided in Methods, and also are available from the corresponding author upon request. Source Data are provided with this paper.

Field-specific reporting

Please select the one below that is the best fit for your research. If you are not sure, read the appropriate sections before making your selection.

- [x] Life sciences
- [ ] Behavioural & social sciences
- [ ] Ecological, evolutionary & environmental sciences

For a reference copy of the document with all sections, see nature.com/documents/nr-reporting-summary-flat.pdf

Life sciences study design

All studies must disclose on these points even when the disclosure is negative.

Sample size	Sample size was not determined a priori. Sample size was limited by availability of research animals. It was set to be no less than 4 animals per group per time point, which is considered to be acceptable practice in the field.
Data exclusions	No data were excluded from the analysis.
Replication	To collect data in all experiments, we infected no less than 4 animals per group with each of the viruses used in this study. For studies depicted in Fig 1c, 3, 4, and 5 we performed no less than 2 independent experimental infections of animals in groups of no less than 4 animals per group. We confirm that all replications generated similar results. For data sets depicted in Fig 2, 6, and 7 one experiment was performed, and findings were confirmed using different methods. For studies depicted in Fig 1d-1g one or two independent experimental infections of mice were performed depending on mice availability.
Randomization	Mice were randomly allocated to experimental groups.
Blinding	Investigators were not blinded to group allocation during samples collection or data analysis. According to the institutional policies any cage which houses animals infected with human pathogen (ZIKV) should be provided with a label which indicates a specific virus name used in the experiment. All parameters which were measured in this study (viremia, viral load in mouse organ, neutralizing antibody titer, number and distribution of barcode sequences) are objective and did not require subjective judgment or interpretation.

Reporting for specific materials, systems and methods

We require information from authors about some types of materials, experimental systems and methods used in many studies. Here, indicate whether each material, system or method listed is relevant to your study. If you are not sure if a list item applies to your research, read the appropriate section before selecting a response.

Materials & experimental systems

n/a	Involved in the study	
☐	☒	Antibodies
☐	☒	Eukaryotic cell lines
☒	☐	Palaeontology and archaeology
☐	☒	Animals and other organisms
☒	☐	Human research participants
☒	☐	Clinical data
☒	☐	Dual use research of concern

Methods

n/a	Involved in the study	
☒	☐	ChIP-seq
☒	☐	Flow cytometry
☒	☐	MRI-based neuroimaging

Antibodies

Antibodies used	Anti-Zika virus NS2B antibody (1:500 dilution factor; GTX133308, GeneTex). We used anti-rabbit Poly-HRP-IgG secondary antibodies containing in the Bond Polymer Refine Detection Kit (DS9800, Leica).
Validation	Validation of anti-Zika virus NS2B antibody was performed using immunofluorescent analysis against ZIKV which was reported in manufacturer's website (https://www.genetex.com/Product/Detail/Zika-virus-NS2B-protein-antibody/GTX133308)

Eukaryotic cell lines

Policy information about cell lines

Cell line source(s)	1) Vero (African green monkey kidney) cells were obtained from World Health Organization.<br>2) DC2 cells (immortalized mouse distal caput epididymal epithelial cells; cat# T059, Applied Biological Materials Inc.).
Authentication	None of the cell lines used were authenticated
Mycoplasma contamination	Cells were not tested for Mycoplasma contamination

Commonly misidentified lines (See ICLAC register)	None of the commonly misidentified cells lines were used in this study

Animals and other organisms

Policy information about studies involving animals; ARRIVE guidelines recommended for reporting animal research

Laboratory animals	10-20 week old male and female AG129 mice, and 10 week old CD1 female mice (Mus musculus).
Wild animals	Study did not involve wild animals.
Field-collected samples	Study did not use samples collected from the field
Ethics oversight	All animal study protocols were approved by the NIAID/NIH Institutional Animal Care and Use Committee (IACUC) and performed in compliance with the guidelines of the NIAID/NIH IACUC. The NIAID DIR Animal Care and Use Program acknowledges and accepts responsibility for the care and use of animals involved in activities covered by the NIH IRP's PHS Assurance D16-00602, last approved 6/10/2019.

Note that full information on the approval of the study protocol must also be provided in the manuscript.
